# Glycoprotein IIb/IIIa inhibitors for cardiogenic shock complicating acute myocardial infarction: a systematic review, meta-analysis, and meta-regression

**DOI:** 10.1186/s40560-020-00502-y

**Published:** 2020-11-11

**Authors:** Carolina Saleiro, Rogério Teixeira, Diana De Campos, João Lopes, Bárbara Oliveiros, Marco Costa, Lino Gonçalves

**Affiliations:** 1 Serviço de Cardiologia, Centro Hospitalar e Universitário de Coimbra, Quinta dos Vales, 3041-801 Coimbra, Portugal; 2 Coimbra Institute for Biomedical Research, Coimbra, Portugal; 3 Faculdade de Medicina da Universidade de Coimbra, Coimbra, Portugal

**Keywords:** Glycoprotein IIb/IIIa inhibitors, Abciximab, Eptifibatid, Cardiogenic shock

## Abstract

**Background:**

Cardiogenic shock complicates 5–10% of myocardial infarction (MI) cases. Data about the benefit of glycoprotein IIb/IIIa inhibitors (GPI) in these patients is sparse and conflicting.

**Methods:**

We performed a systematic review, meta-analysis, and meta-regression of studies assessing the impact of GPI use in the setting of MI complicated cardiogenic shock on mortality, angiographic success, and bleeding events. We systematically searched for studies comparing GPI use as adjunctive treatment versus standard care in this setting. Random-effects meta-analysis and meta-regression were performed.

**Results:**

Seven studies with a total of 1216 patients (GPI group, 720 patients; standard care group, 496 patients) were included. GPI were associated with a 45% relative reduction in the odds of death at 30 days (pooled OR 0.55; 95% CI 0.35–0.85; *I*^2^ = 57%; *P* = 0.007) and a 49% reduction in the odds of death at 1 year (pooled OR 0.51; 95% CI 0.32–0.82; *I*^2^ = 58%; *P* = 0.005). Reduction in short-term mortality seemed to be more important before 2000, as this benefit disappears if only the more recent studies are analyzed. GPI were associated with a 2-fold increase in the probability of achieving TIMI 3 flow (pooled OR, 2.05; 95% CI 1.37–3.05; *I*^2^ = 37%, *P* = 0.0004). Major bleeding events were not increased with GPI therapy (pooled OR, 1.0; 95% CI 0.55–1.83; *I*^2^ = 1%, *P* = 0.99). Meta-regression identified that patients not receiving an intra-aortic balloon pump seemed to benefit the most from GPI use (*Z* = − 1.57, *P* = 0.005).

**Conclusion:**

GPI therapy as an adjunct to standard treatment in cardiogenic shock was associated with better outcomes, including both short- and long-term survival, without increasing the risk of bleeding.

## Background

Cardiogenic shock complicates 5 to 10% of myocardial infarction (MI) cases [1, 2]. Apart from immediate revascularization, no other treatment has been shown to improve outcomes, and reported intra-hospital mortality ranges from 23 to 44% [3, 4]. Further, optimal treatment options are still debated. These features underscore the difficulty in conducting trials in this setting.

Activation of the platelet glycoprotein IIb/IIIa receptor is the final common pathway in the process leading to platelet aggregation, making glycoprotein IIb/IIIa inhibitors (GPI) the most powerful antiplatelet drugs [5]. Their valuable effects in patients with acute coronary syndrome (ACS) have been shown in several trials. A recent meta-analysis of 21 randomized controlled trials by Karathanos et al. found that routine GPI use in ST-elevation myocardial infarction (STEMI) significantly reduced not only the risk for mortality at 30 days and 6 months but also for recurrent ischemic events [6]. However, this was at the cost of an increased risk for all bleeding outcomes, except for intracranial hemorrhage [6]. Most of the studies included a low percentage of patients with shock and were conducted in the era before routine use of novel oral P2Y12 receptor antagonists [7, 8].

Currently, the 2017 European Society Guidelines for the management of acute STEMI recommend considering GPI as a bailout therapy in the event of angiographic evidence of a large thrombus, slow or no reflow, and other thrombotic complications (recommendation class IIa; level C) [9]. In the setting of MI complicated by cardiogenic shock or after cardiopulmonary resuscitation, the best antiplatelet treatment is not currently known. Acetylsalicylic acid may be given intravenously (IV), but most P2Y12 inhibitors must be given orally and most patients in this setting do not have a patent oral route. Although there is the possibility of crushing or dissolving tablets and administering them via a nasogastric tube, many unstable patients may not be intubated by the time they reach the cath lab. Also, gastroparesis and delayed gut absorption may be expected in such patients [10]. This leads to the theory that adjunctive use of GPI is a good choice in cardiogenic shock patients, mainly because of the IV use, high potency, and rapid onset of action. Currently, data about the benefit of GPI in these patients is sparse and conflicting.

We conducted a review aiming to collect available evidence regarding the use of GPI in the context of acute MI complicated by cardiogenic shock. Also, we conducted a meta-analysis and meta-regression to evaluate the impact of GPI use in these patients on short- and long-term mortality, successful revascularization on angiography, and major bleeding.

## Methods

### Study identification and selection

We searched MEDLINE, Embase, and the Cochrane Library databases using the key terms “glycoprotein IIb/IIIa inhibitors” or “abciximab” or “tirofiban” or “eptifibatide” and “cardiogenic shock” without language or date restriction in April 2020. We also manually searched the references from the articles of interest to identify other potentially relevant studies. This data meta-analysis was conducted in accordance with the PRISMA statement. The papers to be included were selected according to a 3-step methodology: (1) reading the title and evaluating its relevance, (2) reading the abstract, and (3) reading the full text.

Articles were considered for inclusion in the analysis if (1) they included a population of patients with cardiogenic shock complicating acute MI and (2) had available data regarding comparisons between groups treated with standard care with adjunctive GPI use and standard care without GPI and (3) data of at least 30-day or 1-year mortality. No strict definition for cardiogenic shock was considered as an inclusion criteria; rather, it was accepted as defined by each research group of the included studies (see Table 1). Standard care was also accepted as the treatment offered to each individual patient, considering that it was the best treatment option for each unique clinical situation. Major bleeding events were included as defined by each study group and are summarized in Table 1. Observational studies were accepted for inclusion. Exclusion criteria included duplicate publication and studies published only in the form of an abstract or as oral conference presentations. Two authors systematically screened the titles and abstracts of publications retrieved using the search strategy in order to select studies which met the inclusion criteria. Any disagreement between them over the eligibility of studies was resolved through discussion and involvement of a third author.

**Table 1:** Outcomes definition by study included in the meta-analysis

	Cardiogenic shock definition	Bleeding definiton	Antiplatelet therapy
Giri et al. [11]	Persistent systolic BP < 90 mmHg, not responsive to fluid resuscitation, or need for vasopressor agents to maintain a systolic BP > 90 mmHg, with evidence of pulmonary congestion and systemic signs of hypoperfusion.	Not applicable	All patients: aspirin and intravenous heparin. Abciximab was administered in the catheterization laboratory at the discretion of the operator: (0.25 mg/kg bolus followed by a 12-h infusion at 10 ug/min). All patients who received stents were maintained on ticlopidine (250 mg twice daily) or clopidogrel (75 mg once daily) plus aspirin (325 mg once daily) for 21 to 30 days.
Chan et al. [12]	Cardiac index ≤ 2.2 L/min/m^2^ with left ventricular end-diastolic pressure > 18 mmHg or persistent (> 30 min) systolic BP > 80 mmHg associated with elevated neck veins	Not applicable	Not specified
Antoniucci et al. [13]	Systolic BP < 90 mmHg (without inotropic or intra-aortic balloon support) associated with signs of end-organ hypoperfusion. Confirmed by cardiac catheterization by systolic BP < 90 mmHg and left ventricular filling pressure > 20 mmHg.	Not specified	Abciximab was administered immediately before the procedure (0.25 mg/kg bolus followed by a 12-h infusion at 10 ug/min). Aspirin (325 mg/day indefinitely) and ticlopidine (500 mg/day for 1 month).
Tousek et al. [14]	At least one of the following criteria: (1) shock index > 1, i.e., sustained hypotension (systolic BP < 90 mmHg) and HR > 90/min; (2) organ hypoperfusion, cold, wet, sweating skin, and HR > 90/min; (3) the need for catecholamine support to maintain BP > 90/min; or (4) Killip class II–III and systolic BP < 120 mmHg	TIMI criteria as major bleeding (intracranial, overt bleeding with a decrease in hemoglobin > 5 g/l, or decrease in hematocrit > 15%)	All patients received standard antithrombotic and anticoagulant treatment during transport/ at the catheterization laboratory (heparin, aspirin and 300-600 mg of clopidogrel). Patients randomized to receive an intravenous bolus of 0.25 mg/kg abciximab after randomization (upfront administration), followed by the infusion of 0.125 μg/kg/min abciximab (maximum 10 μg/min) for 12 h
Bernat et al. [15]	Systolic BP ≤ 90 mmHg for at least 30 min, HR ≥ 60/min, signs of systemic hypoperfusion, or required inotropes to maintain a systolic BP ≥ 90 mmHg.	Not applicable.	Not specified.
Felice et al. [16]	Systolic BP < 90 mmHg for > 30 min or requiring inotropes to maintain systolic BP > 90 mmHg, evidence of low cardiac output with end organ hypoperfusion and signs of elevated filling pressure (e.g., pulmonary congestion on physical examination or chest X-ray).	Intracranial or clinically significant (drop in hemoglobin > 5 g/dl).	All patients were pre-treated with i.v. aspirin 300–500 mg and 300 or 600 mg clopidogrel. i.v. abciximab: pre-procedural bolus of 0.25 mg/kg followed by continuous infusion of 0.125 μg/kg/min for 12 h (up to a maximum dose of 10 ug/min). Aspirin (100 mg) indefinitely and clopidogrel (75 mg) daily for 6–12 months.
Kanic et al. [17]	Hypotension (systolic BP < 90 mmHg for > 30 min or the need for supportive measures to maintain systolic BP > 90 mmHg) and evidence of end-organ hypoperfusion. Also included patients after CPR on admission.	According to TIMI bleeding criteria.	Aspirin 300–500 mg orally or 300 mg i.v. and a 300–600 mg clopidogrel were administered until 2011, after which 60 mg prasugrel or 180 mg ticagrelor were mostly used. Upstream administration of P2Y12 was at the discretion of the emergency physician. GPI (eptifibatide or abciximab) use was at the discretion of the operator (not used upstream).

*BP* blood pressure, *HR* heart rate, *i.v.* intravenous, *TIMI* thrombolysis in myocardial infarction, *CPR* cardiopulmonary resuscitation

**Fig. 1:**
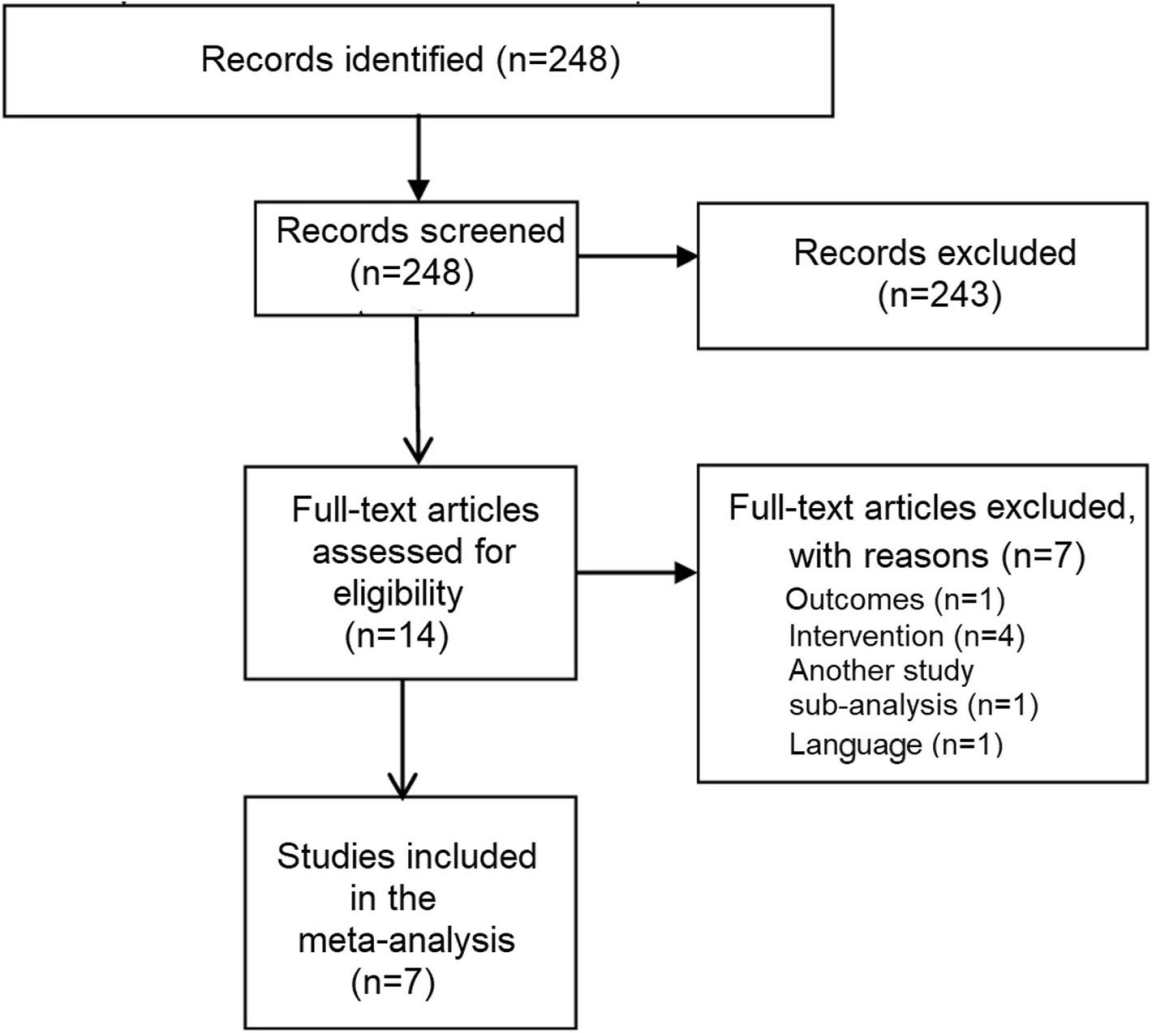
Study identification and selection diagram

A total of 248 studies were identified. Of these, 234 were excluded after title/abstract analysis, as it was evident that they did not fulfill the inclusion criteria or contained duplicate findings. Four studies were excluded after complete analysis because there was not enough data to conduct our analysis or there was no direct comparison between groups of interest. One study was excluded because it was a sub-analysis of another included trial, and another was excluded because it showed only intra-hospital mortality. Another was excluded because the full text was available only in Russian. The selection diagram is shown in Fig. 1. Study design and characteristics were collected from all studies included in the analysis. Data regarding age, gender, hypertension, diabetes mellitus, tobacco use, previous MI, 3-vessel disease, left main disease, left ventricular ejection fraction (LVEF), invasive mechanical ventilation, intra-aortic balloon pump (IABP), and thrombolysis in myocardial infarction (TIMI) flow pre- and post-procedure were considered relevant for cohort characterization and were also collected, when available.

The primary endpoint was 30-day mortality. Secondary endpoints were 1-year mortality, successful revascularization on angiography, and major bleeding. The impact of age, gender, hypertension, diabetes mellitus, tobacco use, mechanical ventilation, LVEF, TIMI flow 0/1 pre-procedure, IABP pump use, or left main lesion on 30-day mortality between groups was analyzed by a meta-regression.

Cardiogenic shock and major bleeding definition, as well as antiplatelet therapy by study included, is shown in Table 1.

### Risk of bias assessment

**Fig. 2:**
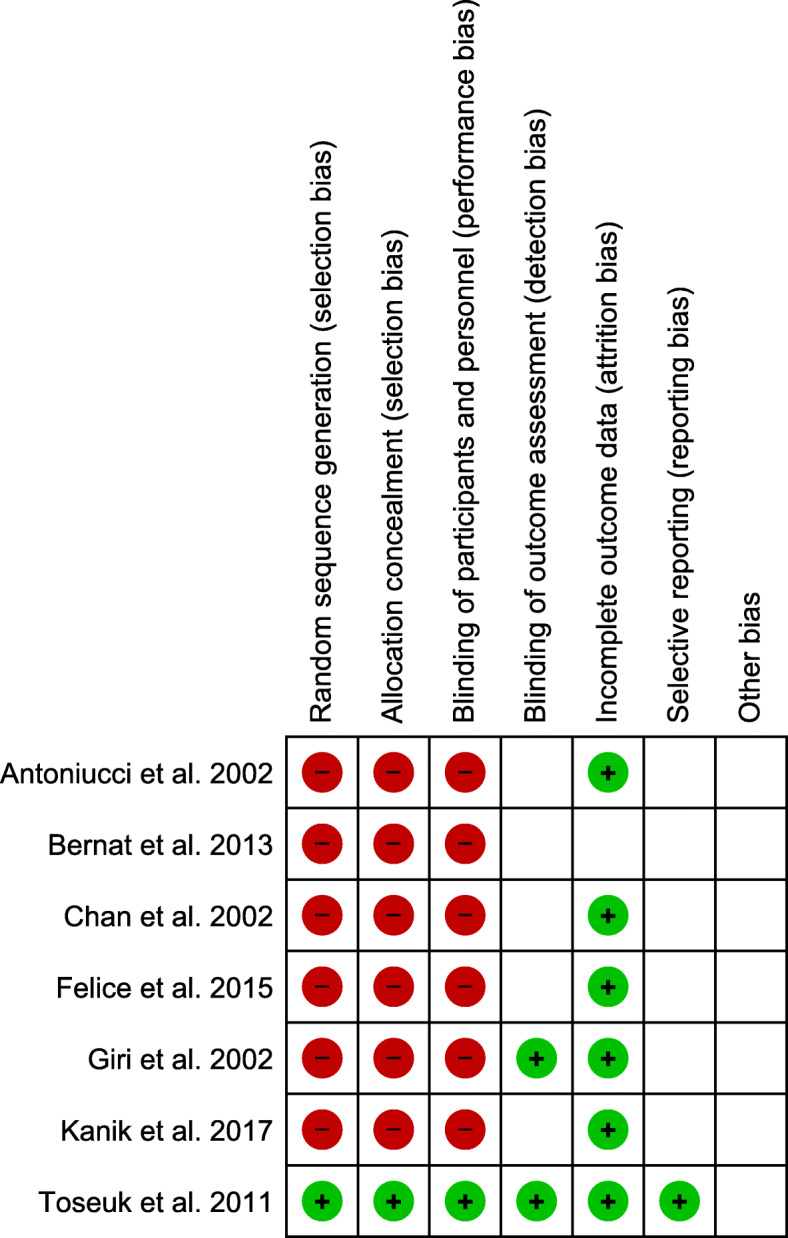
Risk of bias summary. Red, high risk of bias; blank space unclear risk of bias; green, low risk of bias

Two authors independently assessed the risk of bias of the included articles, following the Cochrane Collaboration’s “Risk of bias” tool. Studies were assessed as “low,” “high,” or “unclear” risk for the following biases: random sequence generation, allocation concealment, blinding of participants and personnel, blinding of outcome assessment, incomplete outcome data, and selective reporting. The quality assessment for each study is presented in the “risk of bias summary” (Fig. 2).

### Statistical analysis

Continuous variables are expressed as mean ± standard deviation for normally distributed data or median and interquartile range for non-normally distributed data, and categorical variables are expressed as frequencies or percentages. Pooled odds ratios (OR) and 95% confidence intervals (CI) were estimated based on a random effects meta-analysis and were obtained from the pooled adjusted OR of primary studies. Statistical significance was accepted for *P* values < 0.05. The *I*^2^ statistic was used to assess statistical heterogeneity across studies (moderate heterogeneity was considered present for values between 30 and 60%). Meta-regression was conducted according to the mixed-effects model. We used Review Manager 5.2 (Nordic Cochrane Centre, London, UK) software to perform statistical analyses. The meta-regression was performed using R software through R Studio, version 1.1.463 (R Foundation for Statistical Computing, Vienna, Austria).

## Results

### Studies for the analysis

Seven studies that comprised a total of 1216 patients were included in the analysis (720 patients in the GPI group and 496 patients in the standard treatment group). In the GPI group, 66% of the patients received abciximab and 22% received eptifibatide; for 12% of the patients, the GPI received was not reported. Only 1 study was a randomized trial [14]; all the others had an observational design [11–13, 15–17]. The details of the studies included in the analysis are displayed in Table 2.

**Table 2:** Studies included in the analysis and main findings

Study	Design	No. patients	GPI (***n***)	Age (GPI vs Standard care)	Outcomes
Giri et al. [11]	Nonrandomized, prospective, observational (1995–1999)	114	54	66 ± 14 vs 67 ± 13	• GPI significantly improved final TIMI flow. • At 30-day follow-up, the composite event rate of death, myocardial reinfarction, and target vessel revascularization were better in the GPI group (31% vs 63%; *P* = 0.002). • Lower mortality (22% vs 44%; *p* = 0.02) and recurrent infarction rates (0% vs 10%; *p* = 0.05) in the GPI group. • Combination of abciximab and stents was synergistic and resulted in improvement of all components of the composite end point.
Chan et al. [12]	Nonrandomized, prospective, observational (1993–2000)	96	45	67 ± 11 vs 64 ± 14 vs 63 ± 16 vs 68 ± 9 (Stent+abciximab vs stent only vs PCI + abciximab vs PCI alone)	• 2.5 years of follow-up, the mortality rates for stent plus abciximab, stent only, PCI plus abciximab, and PCI alone were 33%, 43%, 61%, and 68% (*P* = 0.028). • Stent and abciximab resulted in higher TIMI 3 flow rates (85% vs 65%, *P* = 0.048). • A trend of mortality benefit with abciximab was seen at 30 months (HR 0.74, 95% CI 0.36–1.11, *P* = 0.11).
Antoniucci et al. [13]	Nonrandomized, prospective, observational (1999–2001)	77	44	66 ± 12 vs 71 ± 12	• 1-month overall mortality rate was lower in the abciximab (18% vs 42%, *P* < 0.020). • No differences between groups in reinfarction and target vessel revascularization rates. • Multivariate analysis showed that abciximab therapy was the only variable independently related to 1-month mortality (OR 0.36; 95% CI 0.15–0.86, *P* < 0.021).
PRAGUE-7 Tousek et al. [14]	Randomized control trial (2006–2009)	80	40	64 ± 14 vs 68 ± 11	• The 30-day combined outcome of death/reinfarction/stroke/new severe renal failure occurred in 42.5% in the group randomized to abciximab vs 27.5% in the standard therapy group (*P* = 0.24). • No differences were found in TIMI-flow after PCI or major bleeding.
Bernat et al. [15]	Nonrandomized, observational (2006–2010)	179	80	NA	• The use of GPI (HR 0.63, 95% CI 0.40–0.96, *P* = 0.032) was associated with a better outcome at a-year in multivariable analysis.
Felice et al. [16]	Nonrandomized, observational (2002–2011)	410	287	67 ± 12 vs 73 ± 12	• No difference at a 1-year survival rates (42.8% vs 51.6%, *P* = 0.56) in patients treated without vs. with abciximab. • Age, oro-tracheal intubation, post-PCI TIMI flow grade 0–1 but not abciximab use (HR, 1.08; 95% CI 0.70–1.60, *P* = 0.60) were independent predictors of death at 1-year follow-up.
Kanic et al. [17]	(2009–2014)	261	170	66.7 ± 12.5 vs 64.9 ± 12.9	• All-cause mortality was similar between groups (46.5% vs 54.9%) at 30 days; (53.5% vs. 61.1%) at 1 year. • The adjunctive usage of GPI (OR 0.41; 95%CI 0.20–0.84; *P* = 0.015), and novel P2Y12 inhibitors were associated with a better 30-day survival. • Better 1-year survival was independently predicted by GPI (HR 0.62; 95%CI 0.39–0.97; *P* = 0.037), novel P2Y12 inhibitors, age, left main PCI, TIMI 0/1 pre-PCI, and major bleeding.

*GPI* glycoprotein IIb/IIIa inhibitors, *TIMI* thrombolysis in myocardial infarction, *PCI* percutaneous coronary intervention, *NA* not applicable, *HR* hazard ratio, *OR* odds ratio, *CI* confidence intervals

### Baseline characteristics

The mean age of the study population was similar between groups (66.6 ± 12.6 years in the GPI group vs. 69.1 ± 12.3 years in the standard treatment group). There were more males and tobacco users in the standard treatment group, and there were more hypertensive patients in the GPI group. No other important differences between groups were noted. The baseline characteristics are listed in Table 3.

**Table 3:** Baseline characteristics

**Analysis**	**GPI**	**Standard treatment**	**MD**	**95% CI**	***P*** **value**	***I***^**2**^ **(%)**
**Age**	66.6 ± 12.6	69.1 ± 12.3	− 2.75	− 0.64 to 0.83	0.13	74
**LVEF**	35 ± 9	32 ± 10	0.33	− 1.83 to 2.49	0.63	27
**Analysis**	**GPI**	**Standard treatment**	**OR**	**95% CI**	***P*** **value**	***I***^**2**^ **(%)**
Gender (male)*	470/640	258/397	1.43	1.09–1.89	0.01	8
**Diabetes mellitus**	112/470	90/306	0.77	0.55–1.08	0.13	0
Hypertension*	247/425	168/225	0.68	0.49–0.94	0.02	74
Tobacco use*	197/470	87/306	1.87	1.20–2.91	0.006	36
**Previous MI**	82/470	77/306	0.75	0.48–1.18	0.21	25
**3 vessel disease**	181/425	124/225	0.64	0.29–1.45	0.29	79
**Left main lesion**	74/446	31/298	1.54	0.52–4.57	0.44	76
**Invasive ventilation**	312/586	198/338	1.20	0.62–2.23	0.57	65
**IABP**	363/640	149/397	2.13	0.96–4.72	0.06	82
**TIMI 0/1 pre-procedure**	167/595	185/346	0.60	0.19–1.88	0.38	90

Bernat et al.’s study did not provide information regarding baseline data and was therefore not included in the baseline characteristics analysis

*GPI* glicoprotein IIb/IIIa inhibitors, *MD* mean difference, *CI* confidence intervals, *OR* odds ratio, *LVEF* left ventricular ejection fraction, *MI* myocardial infarction, *IABP* intraortic balloon pump, *TIMI* thrombolysis in myocardial infarction

### Short-term (30-day) mortality

**Fig. 3:**
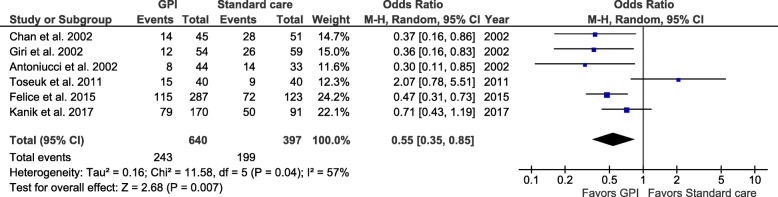
Pooled analysis for 30-day mortality comparing GPI and standard care. Numbers displayed represent ORs with 95% CIs. CI, confidence interval; GPI, glycoprotein IIb/IIIa inhibitors; OR, odds ratio

**Fig. 4:**
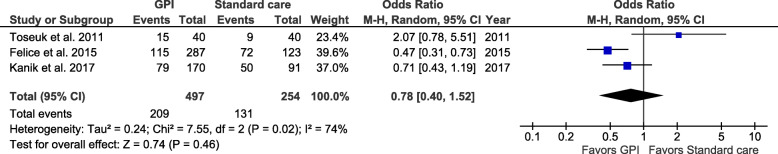
Sensitivity analysis for 30-day mortality comparing GPI and standard care. Numbers displayed represent ORs with 95% CIs. CI, confidence interval; GPI, glycoprotein IIb/IIIa inhibitors; OR, odds ratio

All studies were primarily designed to evaluate the use of GPI on 30-day mortality: a total of 1037 patients were evaluated for this endpoint. In all, 37% of patients were dead at 30 days in the GPI group compared to 50% in the standard care group. GPI use was associated with a significant 45% relative reduction in the odds of death (Fig. 3) (OR 0.55; 95% CI 0.35–0.85; *I*^2^ = 57%; *P* = 0.007). However, there was moderate heterogeneity between studies with respect to outcomes (*I*^2^ = 57%; *P* = 0.04). In a sensitivity analysis, after excluding studies that included patients until 2000 [11–13], resulting in a total of 751 patients and 340 pooled events, GPI use was no longer associated with 30-days mortality (Fig. 4) (OR 0.78; 95% CI 0.40–1.52; *I*^2^ = 74%; *P* = 0.46). There was still substantial heterogeneity between studies with respect to the outcome (*I*^2^ = 74%; *P* = 0.02).

Meta-regression for age, gender, hypertension, diabetes mellitus, tobacco use, mechanical ventilation, LVEF, TIMI flow 0/1 pre-procedure, or left main culprit lesion revealed no statistically significant differences (Table 4). Only the inclusion of the IABP variable in the meta-regression influenced the outcome (*Z* = − 1.57, *P* = 0.005) (Table 4), and GPI use was associated with a higher OR when patients did not receive an IABP.

**Table 4:** Meta-regression for 30-day mortality

	***n***	***I***^**2**^	Q_**df**_ (***p***)	Coef (***p***)
**Age**	5	61.32%	7.32_3_ (0.062)	− 0.307 (0.210)
**Gender**	6	51.72%	8.72_4_ (0.068)	4.475 (0.169)
**Diabetes mellitus**	6	68.09%	10.42_4_ (0.034)	− 0.849 (0.708)
**Hypertension**	3	85.10%	6.71_1_ (0.010)	6.832 (0.387)
**Tobacco use**	4	81.47%	8.111_2_ (0.017)	6.774 (0.456)
**Invasive ventilation**	5	77.08%	10.614_3_ (0.014)	− 0.165 (0.893)
**TIMI flow 0/1 pre procedure**	5	77.28%	10.681_3_ (0.014)	− 0.288 (0.807)
IABP use*	6	0.00%	3.762_4_ (0.439)	− 1.565 (0.005)
**LVEF**	3	0.00%	0.002_1_ (0.989)	− 0.665 (0.368)
**Left main stenosis**	4	41.01%	3.279_2_ (0.194)	− 15.572 (0.851)

*n* number of studies included in the analysis, *TIMI* thrombolysis in myocardial infarction, *IABP* intraortic balloon pump, *LVEF* left ventricular ejection fraction

### Long-term mortality

**Fig. 5:**
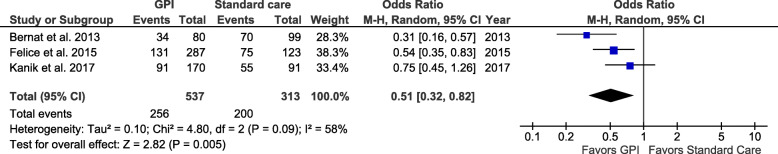
Pooled analysis for 1-year mortality comparing GPI and standard care. Numbers displayed represent ORs with 95% CIs. CI, confidence interval; GPI, glycoprotein IIb/IIIa inhibitors; OR, odds ratio

Three observational studies including 850 patients reported 1-year mortality. Mortality was 47% in the GPI group and 60% in the standard care group. The pooled analysis (Fig. 5) showed a 49% reduction in the odds of 1-year mortality in favor of the GPI group (OR 0.51; 95% CI 0.32–0.82; *I*^2^ = 58%; *P* = 0.005).

### Successful revascularization: TIMI 3 flow after PCI

**Fig. 6:**
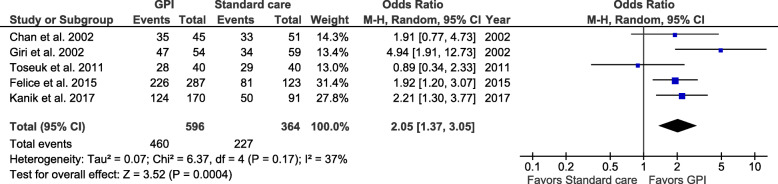
Pooled analysis for TIMI 3 flow after PCI comparing GPI and standard care. Numbers displayed represent ORs with 95% CIs. CI, confidence interval; GPI, glycoprotein IIb/IIIa inhibitors; OR, odds ratio; TIMI, thrombolysis in myocardial infarction. Please note that for easy understanding “standard care” is represented “left” and “GPI” is presented “right” in the forest plot

**Fig. 7:**
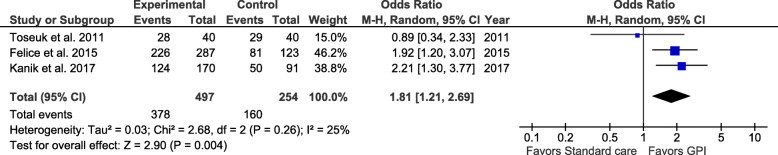
Sensitivity analysis for TIMI 3 flow after PCI comparing GPI and standard care. Numbers displayed represent ORs with 95% CIs. CI, confidence interval; GPI, glycoprotein IIb/IIIa inhibitors; OR, odds ratio; TIMI, thrombolysis in myocardial infarction. Please note that for easy of understanding “standard care” is represented “left” and “GPI” is presented “right” in the forest plot

TIMI 3 flow after PCI was achieved in 84% of the patients in the GPI group and 70% in the standard care group. The pooled data showed that adjunctive use of GPI was associated with a 2-fold increase in the probability of achieving TIMI 3 flow after PCI (Fig. 6) (OR 2.05; 95% CI 1.37–3.05; *I*^2^ = 37%, *P* = 0.0004). Antoniucci et al. reported only data regarding successful angioplasty (not specific for TIMI 3 flow). When this study was included in the analysis, the results were similar to those presented for TIMI 3 flow (data not shown). In a sensitivity analysis, after excluding studies that included patients until 2000 [11, 12], GPI use is still associated with a higher odds of achieving TIMI 3 flow (Fig. 7) (OR 1.81; 95% CI 1.21–2.69; *I*^2^ = 25%, *P* = 0.004). This sensitivity analysis reduced heterogeneity between studies with respect to outcomes (*I*^2^ = 0.25, *P* = 0.26).

### Safety endpoints: major bleeding events

**Fig. 8:**
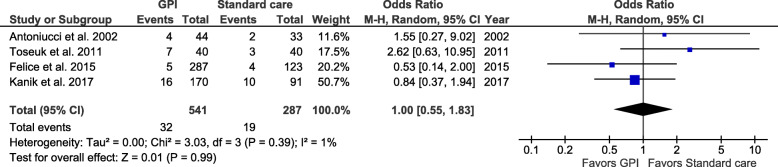
Pooled analysis for major bleeding comparing GPI and standard care. Numbers displayed represent ORs with 95% CIs. CI, confidence interval; GPI, glycoprotein IIb/IIIa inhibitors; OR, odds ratio

**Fig. 9:**
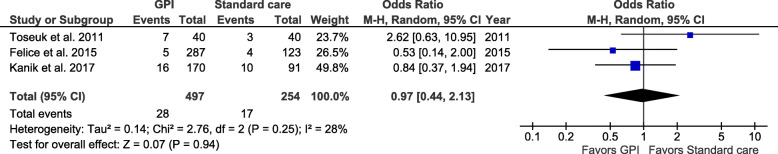
Sensitivity for major bleeding comparing GPI and standard care. Numbers displayed represent ORs with 95% CIs. CI, confidence interval; GPI, glycoprotein IIb/IIIa inhibitors; OR, odds ratio

Bleeding events were reported in 4 studies involving a total of 829 patients (541 treated with GPI). The risk of major bleeding was not significantly increased with GPI therapy compared with standard treatment (Fig. 8) (OR, 1.0; 95% CI 0.55–1.83; *I*^2^ = 1%, *P* = 0.99). In a sensitivity analysis, after excluding studies that included patients until 2000, GPI use did not increase the risk of bleeding (Fig. 9) (OR 0.97; 95% CI 0.44–2.13; *I*^2^ = 28%, *P* = 0.94).

## Discussion

Our meta-analysis showed that among patients with cardiogenic shock complicating MI, GPI adjunctive use may be both effective and safe. Overall, 30-day and 1-year mortality were both almost halved with the use of GPI compared to standard treatment only. The cohort of patients not receiving an IABP benefited the most from GPI use. However, this reduction in short-term mortality seemed to be more important before 2000, as this benefit disappeared if only the more recent studies are analyzed. GPI adjunctive treatment was similarly associated with angiographic success: there was a 2-fold increase in the odds of achieving a TIMI 3 flow after PCI in this group. Also, GPI use adjunctive to standard care showed a good safety profile as it did not increase the risk for major bleeding events.

All studies except one (Prague-7) were observational in nature. In these studies, GPI use and revascularization strategy were at the discretion of the operator and varied depending on time and setting. Older studies included a low number of patients with PCI with stent implantation [11–13]; as in one study, balloon only angioplasty accounted for 57% of all PCIs [11]. Most of the dual antiplatelet therapy consisted of ticlopidine or clopidogrel plus aspirin [11–13]. Until 2011, anti-platelet loading doses consisted of aspirin and/or clopidogrel [16, 17]. More recently, prasugrel or a loading dose of ticagrelor was mostly used concomitantly with aspirin. Abciximab was administered as a bolus followed by continuous infusion. Only a small proportion of patients were treated with a GPI other than abciximab [15, 17]. The Prague-7 trial differed from these observational studies in the fact that a group was randomized to receive upfront administration of abciximab adjunctive to standard therapy, while in the control group, abciximab was administered if deemed necessary by the operator [14]. This study included not only patients in cardiogenic shock but also patients at risk for evolving to cardiogenic shock; in fact, only 47 of the 80 patients included were considered class Killip-Kimbal IV [14]. Kanic et al. also included patients resuscitated from sudden death (161 out of 261 patients), not only patients in cardiogenic shock [17]. See Table 1 for further details. Despite varying cardiogenic shock definitions, all patients included were considered critically ill.

An analysis from 2005 to 2013 showed a reduction in the use of GPI from 76 to 53% [3], and another study showed a similar decrease from 93% in 2010 to 44% in 2014 [17]. Bivalirudin and novel P2Y12 inhibitors (prasugrel/ticagrelor) were used less often in the GPI group. However, in a multivariable analysis, the year of admission was not a predictor of worse outcome [17]. Younger patients without the need for IABP support or orotracheal intubation were more likely to receive abciximab treatment [16]. No differences between groups regarding invasive ventilation and IABP support were noted. Finally, we cannot make assumptions regarding the standard medical therapy as it was not detailed in any of the included studies.

Antoniucci et al. suggested that the clinical benefit of GPI was not related to the patency of the infarct-related artery, as there was no benefit in events related to vessel reocclusion such as reinfarction or need for repeat revascularization. Moreover, most deaths were due to refractory ventricular failure, regardless of a patent vessel [13]. It was hypothesized that abciximab prevents recurrent MI not only via its potent antiplatelet activity but also due to its possible anti-inflammatory effects [18], allowing rapid recovery of coronary vascular function in the microcirculation [19]. The positive effect on coronary microvasculature is mediated by the inhibition of direct interaction of platelets and leukocytes with reperfused endothelium, along with a diminished distal embolization of platelet aggregates [18] Abciximab also permits a more rapid recovery of regional wall motion and ventricular function [19]. This is critical in cardiogenic shock patients and may, in part, explain the better outcomes in mortality that we found.

Despite our results, it is difficult to unreservedly support the routine adjunctive use of GPI in cardiogenic shock patients in the era of novel P2Y12 inhibitors and new revascularization strategies, as they were underrepresented in this meta-analysis. We sought to overcome this handicap by performing a sensitivity analysis excluding studies that included patients before 2000. We chose this timeline because of the publication of the SHOCK trial in 1999 [20]. This trial was the first prospective randomized study in cardiogenic shock. Emergency early revascularization with coronary artery bypass graft or PCI was compared with a strategy of initial medical stabilization with drug therapy and IABP. Mortality rate at 6 months was significantly improved in the early revascularization group, although no difference was noted on 30-day mortality [20]. It had strong impact on clinical care in MI complicated by cardiogenic shock as early revascularization was not clinical standard care before that [20]. In fact, the advantage conferred by GPI in short-term mortality disappeared after exclusion of older studies, while maintaining long-term survival benefit. Still, the IV use of GPI and their rapid onset of action may denote an important advantage for their use in this setting. The new IV P2Y12 inhibitor cangrelor is showing good results in patients with cardiogenic shock, but much still needs to be clarified about its use [21].

### Impact of GPI use on mortality

Concordant with our results showing a positive impact on prognosis, four other studies found that abciximab therapy improves the 30-day outcome of primary PCI in cardiogenic shock [11–13, 17]. Other studies, which were not included in the analysis, also support the benefit of adjunctive use of GPI on in-hospital [22] and 30-day mortality [23]. In a larger study, however, abciximab use was not a predictor of death after multivariable adjustment [16]. The only randomized controlled trial included in the current analysis (Prague-7 study) did not show benefit from routine pre-procedural abciximab when compared with selective use (35% of the patients in the control group) during intervention [14]. Many factors may contribute to this. First, the study allowed the inclusion of patients not only in cardiogenic shock, but also at risk for cardiogenic shock. Second, it tested the routine upfront use of abciximab. Third, the selective use of abciximab, when deemed necessary by the operator, was allowed in the control group. Similarly, the ADMIRAL trial did also not show benefit of GPI use in cardiogenic shock on the combined outcome of death, reinfarction, or urgent target-vessel revascularization [8]. Regarding long-term outcomes, although 1 study showed abciximab use was not an independent predictor of death at 1-year follow-up [16], 2 others showed results consistent with better survival at 1-year predicted by GPI [15, 17].

Overall, 30-day and 1-year mortality were both almost halved in the GPI group in our meta-analysis. This may be explained, in part, by GPI’s high potency, rapid onset of action, and IV route of administration: these characteristics offer it a special role in unstable patients. Until now, the most common P2Y12 inhibitors, except for cangrelor, have been available only in pill form. Hence, in the context of shock, many patients may not have a patent oral route and, even if they do, gastroparesis and delayed absorption may impair the efficacy of these antiplatelet drugs; additionally, these drugs usually have a longer time to platelet inhibition onset, even in stable patients. A report showed that in comatose patients after cardiac arrest undergoing PCI, clopidogrel loading did not significantly affect platelet function during the first 48 h. This contrasted with eptifibatide, which produced profound platelet inhibition [24].

### Angiographic success: TIMI 3 flow after PCI

Our results showed that GPI use adjunctive to standard therapy was associated with a 2-fold increase in the probability of achieving TIMI 3 flow after PCI. Except for the Prague-7 trial, which found no differences in TIMI flow after PCI between groups [14], all studies reporting this data are concordant with our results (73–79% in the GPI group vs. 55–65% in the standard care group) [11, 12, 16, 17]. These results reinforce the efficacy of GPI in this setting, since most of the studies included patients whose GPI was prescribed at the discretion of the operator. This probably means, according to the actual guideline recommendations, that most of the patients receiving GPI had a higher thrombotic load and, even so, TIMI 3 flow after PCI was more likely in this group.

### Safety endpoints: effect on major bleeding

In our meta-analysis, GPI showed a good safety profile. Despite differing criteria for definitions of major bleeding, reports of major bleeding varied from 1 to 10% in the GPI group and 3 to 12.5% in the standard treatment group [13, 14, 16, 17]. There was consistency between studies that GPI use did not increase the risk of major bleeding compared to standard treatment [13, 14, 16, 17]. Although theoretically GPI adjunctive use could contribute to increased bleeding risk, an important consideration may be the fact that most of the studies included patients treated with GPI at the discretion of the operator. In our meta-analysis, no difference between groups regarding age was noted; other important bleeding risk factors and comorbidities may have varied between groups and were probably important considerations in the operators’ decisions. This may induce a selection bias for which we were not able to control. Also, minor differences in major bleeding definitions varied between studies and minor bleedings were not reported in all the included studies.

### Limitations

Most of the studies included had an observational design, and there were also some minor differences in the inclusion criteria for each study, including heterogeneity in the definitions of cardiogenic shock and enrolling patients admitted with cardiac arrest but not shock. Most of the studies were also conducted in the era of clopidogrel and may reflect different standards of care, not only regarding antiplatelet therapy but also revascularization and angioplasty strategies. We sought to overcome some of these limitations by conducting a sensitivity analysis. Whether or not these results would be replicated in the setting of the generalized use of more potent P2Y12 inhibitors with a more rapid onset of action is still unknown.

## Conclusion

GPI therapy as an adjunct to standard treatment in acute MI patients with cardiogenic shock is associated with improved short- and long-term survival without an increased bleeding risk. These results warrant further randomized controlled trials to assess GPI value in these patients and in the context of new revascularization strategies.

## Data Availability

The datasets used and/or analyzed during the current study are available from the corresponding author on reasonable request.

## Abbreviations

MI: Myocardial infarction
GPI: Glycoprotein IIb/IIIa inhibitors
ACS: Acute coronary syndrome
STEMI: ST-elevation myocardial infarction
IV: Intravenous
PCI: Percutaneous coronary intervention
LVEF: Left ventricular ejection fraction
IABP: Intraaortic balloon pump
TIMI: Thrombolysis in myocardial infarction
OR: Odds ratio
CI: Confidence intervals
